# Linarin and Hyperoside Inhibit lptD/msbA to Disrupt Membranes of Multidrug-Resistant *Acinetobacter baumannii*

**DOI:** 10.3390/biology14081087

**Published:** 2025-08-20

**Authors:** Yuqi Yang, Xue Li, Yunshi Chen, Yan Zhang, Lailai Li, Yihui Chai, Xiang Pu, Xin Liu

**Affiliations:** 1 School of Basic Medicine, Guizhou University of Traditional Chinese Medicine, Guiyang 550025, China; yangyuqi061@gzy.edu.cn (Y.Y.); mooching@126.com (L.L.); yihuichai@163.com (Y.C.); puxiang0318@163.com (X.P.); 2 The Second Affiliated Hospital of Guizhou University of Traditional Chinese Medicine, Guiyang 550025, China; lixue199597@163.com (X.L.); 13616224511@163.com (Y.C.); 3 School of Pharmacy, Guizhou University of Traditional Chinese Medicine, Guiyang 550025, China; zhangyan0003@gzy.edu.cn

**Keywords:** multidrug-resistant *Acinetobacter baumannii*, *Senecio scandens*, lptD/msbA, linarin, hyperoside

## Abstract

Against the backdrop of rising multidrug-resistant *Acinetobacter baumannii* (MDR AB) threats, this study explores the in vitro antibacterial activity and mechanism of *Senecio scandens* (a Miao ethnic medicinal herb) crude extract. Using 10 clinical MDR AB strains, we reassessed antibiotic sensitivity and then applied microbroth dilution to determine MIC/MBC, time-kill curves for bactericidal kinetics, and SEM/TEM for structural changes. Proteomics identified downregulated proteins, cross-referenced with VFDB/CARD to target membrane-related proteins (msbA, lptD), while molecular docking validated the strong binding of linarin/hyperoside to these targets. qPCR confirmed lptD/msbA mRNA downregulation (*p* < 0.05) by linarin/hyperoside (MIC = 312.5 μmol/L). The extract showed concentration-dependent bactericidal effects (MIC = 640 μg/mL), disrupting cell wall/membrane integrity. This study first reveals that linarin and hyperoside inhibit MDR AB by downregulating lptD/msbA, compromising outer membrane integrity, offering novel therapeutic candidates.

## Simple Summary

Against the critical challenge of multidrug-resistant *Acinetobacter baumannii* (MDR AB), a pathogen prioritized by the WHO, this study investigates the in vitro antibacterial efficacy and mechanism of *Senecio scandens* (a Miao ethnic medicinal herb) crude extract. Using 10 clinical MDR AB strains, we combined microbroth dilution, time-kill assays, and electron microscopy to demonstrate concentration-dependent bactericidal activity (MIC = 640 μg/mL) via the disruption of cell wall/membrane integrity. Proteomic analysis identified downregulated outer membrane-related proteins (msbA, lptD), validated by molecular docking as targets of linarin/hyperoside (MIC = 312.5 μmol/L). qPCR confirmed significant downregulation of lptD/msbA mRNA (*p* < 0.05), linking flavonoids to outer membrane destabilization. This work first reveals that linarin and hyperoside combat MDR AB by inhibiting essential membrane biogenesis genes, offering novel candidates for natural compounds with antibacterial effects development.

## 1. Introduction

*Acinetobacter baumannii* (*A. baumannii*) is a Gram-negative, non-fermenting coccobacillus that can colonize human skin. It is mostly found in soil and water and is commonly discovered in hospitals and hospitalized patients [1]. Epidemiological investigations have shown that approximately 40% of healthy adults have this bacterium colonized in their skin and mucous membranes. Moreover, the carriage rate is even more pronounced among the medical staff population [1,2]. *A. baumannii* can cause a variety of infectious diseases. It is responsible for respiratory tract infections (e.g., ventilator-associated pneumonia), cutaneous and subcutaneous infections (such as cellulitis), and urinary tract infections in catheterized patients, as well as invasive diseases like meningitis and bacteremia [3,4]. These pose serious risks, especially to immunocompromised patients and those in intensive care. Moreover, its multidrug resistance complicates clinical management and contributes to high morbidity and mortality [4,5]. It is noteworthy that *A. baumannii* is gradually emerging as a significant pathogen in community infections. Community-acquired pneumonia caused by *A. baumannii* often occurs in patients with a history of smoking and diabetes, regardless of whether they have been colonized by this bacterium previously. It is mainly characterized by an acute onset, rapidly progressing to respiratory failure and septic shock, and has an extremely high mortality rate [6,7,8]. In 2024, the WHO still classified carbapenem-resistant *Acinetobacter baumannii* (CR AB) as a critical-priority pathogen [9]. *A. baumannii* possesses multiple types of resistance mechanisms, such as bacterial biofilm formation, multidrug efflux pumps, target alteration, and enzymatic destruction [10,11]. Both pan- and multidrug resistant, *A. baumannii* spreads quickly, is resistant to practically all antibiotics, and is growing into a major worldwide health threat [12]. Therefore, it is crucial to discover and develop novel treatments before microorganisms overcome the antibiotics’ final line of protection.

For thousands of years, Traditional Chinese Herbal Medicine (TCHM), characterized by its multitarget and multi-pathway mechanisms, has been utilized in disease prevention and management. Natural products typically exhibit a multitarget mechanism of action, enabling them to more comprehensively address complex disease states. Relative to certain chemically synthesized drugs, natural products tend to possess lower toxicity and higher safety profiles. For example, methanol and n-hexane extracts of Chenopodium album not only exert antibacterial activity but also contain rich minerals, thereby qualifying as an alternative nutritional therapeutic approach [13]. Furthermore, nanocomposites synthesized via chia seed-mediated processes, in addition to their antibacterial properties, demonstrate diverse bioactivities, including antioxidant, anticancer, and wound-healing-promoting effects [14]. These attributes align with the core tenets of natural product-based therapies, which emphasize holistic regulation and multifunctional treatment. Notably, TCHM has demonstrated efficacy against drug-resistant bacterial infections, as evidenced by multiple studies [15,16,17,18]. Its therapeutic actions operate through several pathways, altering membrane permeability, inhibiting protein and nucleic acid synthesis, impeding in vivo enzyme activity, and restricting the growth potential of pathogenic microorganisms [16]. In the realm of medicinal herbs in Miao ethnic medicine, Qianliguang (senecio scandens Buch.-Ham.) has a long-standing application in the treatment of bacterial infections, predominantly those affecting the respiratory tract and causing bacterial diarrhea, as reported by [19]. Pharmacological studies have revealed that, besides its anti-inflammatory, antibacterial, hepatoprotective, and antiviral properties, senecio scandens harbors potential hepatotoxic and carcinogenic risks [20]. Previous studies have indicated that senecio scandens exhibits relatively low acute toxicity, and its toxicity is associated with factors such as the origin of the medicinal herbs and the extraction methods [21]. The crude extract of senecio scandens is a substance with low toxicity. It can retain the effective components of senecio scandens, including alkaloids and flavonoid compounds, while excluding pyrrolizidine alkaloids, thereby further enhancing the safety profile of senecio scandens [22]. This plant has exhibited broad-spectrum antibacterial efficacy against various pathogens, including β-hemolytic streptococcus, haemophilus influenzae, streptococcus pneumoniae, and staphylococcus aureus [19]. In our previous research, senecio scandens demonstrated potent inhibitory activity against *A. baumannii*, with the minimum inhibitory concentration (MIC) determined to be 640 μg/mL (unpublished data).

This study aims to prepare a crude extract of senecio scandens using an 80% methanol extraction method and systematically evaluate its in vitro antibacterial effect against clinically isolated multidrug-resistant *A. baumannii* (MDR AB). The ultrastructural changes of bacteria after the action of the drug were observed via scanning electron microscopy. In combination with non-targeted proteomics technology, key differentially expressed proteins were screened. Furthermore, computational simulation platforms such as AutoDock Vina were employed to conduct ligand–receptor molecular docking analysis, identify potential active components and their action targets, and verify their interactions through in vitro experiments. This research endeavors to elucidate the molecular mechanism of senecio scandens against MDR AB from the perspectives of protein and gene expression. It aims to provide theoretical support for the development of novel antibacterial drugs and facilitate the translational application of Traditional Chinese Medicine in modern anti-infection therapy.

## 2. Materials and Methods

**Figure 1:**
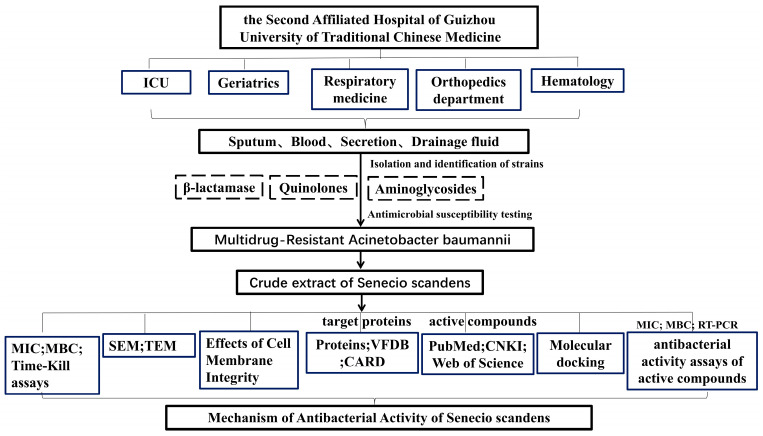
Experimental workflow diagram.

Workflow Diagram: A schematic summarizing the experimental pipeline (MIC/MBC → time kill → SEM/TEM → proteomics → docking/qPCR) is presented in Figure 1.

### 2.1. Drug and Bacterial Isolation

First, 500 g of senecio scandens herbs was extracted three times under reflux with a fivefold volume of 80% methanol for 3 h each. The combined extracts were concentrated into a thick paste using a rotary evaporator and transferred to a beaker. It was then dried in a vacuum drying oven (vacuum degree: −0.1 MPa) at 60 °C for 72 h. After that, it was ground into a uniform powder, dispensed into sterile cryotubes, and stored at −80 °C for the long term.

*Senecio scandens* was prepared in 0.5% DMSO. Colistin (Shanghai Yuanye Bio, Shanghai, China, lot number J11GS151298) was prepared in purified water (Wahaha, Hangzhou, China). Emodin (HY-14393), fisetin (HY-N0182), β-carotene (HY-N0411), quercetin (HY-18085), hyperoside (HY-N0452), and linarin (HY-N0528) (MCE, Trenton, NJ, USA) were prepared in 0.5% DMSO. The stock solutions of the drugs were freshly prepared before each experiment and filtered through a 0.22 μM syringe filter.

The *A. baumannii* strains used in this experimental study were obtained from the Second Affiliated Hospital of Guizhou University of Traditional Chinese Medicine. After being identified by the Clinical Laboratory Department of the hospital, these strains were preserved in our laboratory. The background information of these strains is shown in Table 1. *A. baumannii* ATCC 19606, serving as a control, was purchased from the American Type Culture Collection (ATCC). All strains were inoculated on Muller–Hinson (MH) agar medium (HKM, Guangdong HUANKAI MICROBIAL SCI&TECH. Co., Ltd., Guangzhou, China) and resuscitated in a constant temperature incubator set at 37 °C for a period of 18–24 h. Single uniform and plump colonies were resuspended with normal saline. The samples were then analyzed with a bacterial turbidity meter and subsequently adjusted to the required concentration of 0.5 McFarland standard (1.5 × 10^8^ CFU/mL); during the experiment, the bacterial suspension was diluted to 1.5 × 10^5^ CFU/mL with MH broth.

### 2.2. Determination of the MIC and MBC

This study aims to assess the MIC of colistin and senecio scandens via the twofold broth dilution method in 96-well plates. Initially, 50 μL of MH broth was dispensed into each well, with an additional 50 μL added to the 11th well to serve as a negative control. Subsequently, 50 μL of the colistin or senecio scandens stock solution was introduced into the first well and serially diluted twofold up to the 10th well. Thereafter, a bacterial solution with a concentration of 1.5 × 10^5^ CFU/mL was added to all wells, except the 11th. The 96-well plates were then incubated at 37 °C for 18 ± 2 h. The MIC was defined as the lowest concentration of the antimicrobial agent that completely inhibited visible bacterial growth. To determine the minimum bactericidal concentration (MBC), 100 μL of the suspensions from wells at the MIC and those with drug concentrations higher than the MIC were plated. The MBC was identified as the lowest drug concentration capable of eradicating 99.9% or more of viable microorganisms. All experimental procedures were replicated three times to ensure the reliability and consistency of the results.

### 2.3. Time-Kill Curve Assay

Colistin and senecio scandens were prepared in the MH broth alone. Next, co-cultures were prepared by mixing 100 μL of MDR AB55282 at 1.5 × 10^5^ CFU/mL with varying concentrations of colistin (0.5 μg/mL, 0.25 μg/mL, and 0.125 μg/mL) and senecio scandens (1280 μg/mL, 640 μg/mL, and 320 μg/mL) and then incubated at 37 °C. A total of 10 μL of culture solution was added to 0.99 mL of MH broth (10^−2^ dilution) at 0, 2, 4, 6, 8, 12, and 24 h, and then 10 μL of each dilution was added to 0.99 mL of MH broth for serial dilution to obtain the 10^−4^, 10^−6^, 10^−8^, 10^−10^, and 10^−12^ dilutions. At various intervals, 100 μL of co-culture solution was diluted to each concentration and spread evenly on MH agar medium for bacterial counting after incubating at 37 °C for 24 h.

### 2.4. Scanning Electron Microscopy (SEM) and Transmission Electron Microscopy (TEM)

The time-kill curve procedure was replicated, and the test solution was incubated for the specified duration. Following incubation, the mixture underwent centrifugation at 5000× *g* for 10 min at 4 °C to harvest the bacterial cells. A control group was established following the same protocol, with the exception that no therapeutic drug was added. The collected bacterial cells were first fixed with 3% glutaraldehyde, followed by post-fixation in 1% osmium tetroxide. Subsequently, the samples were dehydrated using acetone and stained with uranyl acetate and citric acid. Microscopic examination was performed using a JSM-IT700HR scanning electron microscope (JEOL Ltd., Akishima, Tokyo, Japan) and a JEM-1400FLASH transmission electron microscope (JEOL Ltd., Akishima, Tokyo, Japan) to visualize the cellular structures.

### 2.5. Effects of Cell Membrane Integrity

The experimental method was carried out according to the previous research [23]. Overnight cultures of MDR AB 55282 in TSB were harvested, washed with sterile 0.9% physiological saline, and subsequently resuspended. The bacterial suspension was adjusted to a turbidity of 0.5 MCF (1.5 × 10^8^ CFU/mL) using a bacterial turbidity meter and was then diluted in MH broth (1:999) to obtain a working concentration of 1.5 × 10^5^ CFU/mL. Test solutions containing colistin (0.5 μg/mL) and senecio scandens (1280 μg/mL) were incubated with bacterial suspensions. The mixtures were incubated in a shaking incubator at 37 °C for 0, 1, 2, 3, and 4 h. After centrifugation (12,000× *g*, 2 min), the supernatant was filtered through a 0.22 μm membrane. The filtrate was analyzed using a microplate reader at 260 nm.

### 2.6. Collection of Active Compounds in Senecio Scandens and Acquisition of Target Proteins

By consulting domestic and international academic databases such as PubMed (https://pubmed.ncbi.nlm.nih.gov/, accessed on 15 August 2025), China National Knowledge Infrastructure (CNKI; https://www.cnki.net/, accessed on 15 August 2025), Web of Science (https://www.webofscience.com/wos/, accessed on 15 August 2025), etc., the relevant literature on the chemical components of senecio scandens were collected using keywords like “Qianliguang”, “*Senecio scandens*”, “Buch.-Ham.”, and “active compounds”. Information on the reported active compounds was obtained from this literature.

The proteins were extracted from the co-cultured bacterial suspension using the SDT (4% SDS, 100 mM Tris-HCl, pH 7.6) lysis method. Subsequently, the protein concentration was determined by the BCA method. Data were collected through LC-MS/MS, and the raw data were subjected to database searching for identification and quantitative analysis using MaxQuant software (version 1.6.10.43). Differential analysis was conducted based on the criteria that the fold change (FC) was >2 or <1/2 and *p* < 0.05.

An intersection was identified between the downregulated differential proteins obtained from proteomic analysis as well as the Virulence Factors Database (VFDB) and the Comprehensive Antibiotic Resistance Database (CARD) to obtain the target sites.

### 2.7. Molecular Docking

The 3D structural files of the active components were obtained in SDF format from the PubChem database (https://pubchem.ncbi.nlm.nih.gov, accessed on 15 August 2025). These files were then converted to PDB format using PyMol 2.4.0. The 3D crystal structures of the target proteins were also retrieved from the PDB database (https://www.pdbus.org/, accessed on 15 August 2025). Subsequently, ions and water molecules were removed from these structures through PyMol 2.4.0, and PDB files were generated. Autodock Tools 1.5.7 software was employed for modifications. The active components and target proteins were separately converted into the pdbqt format of ligands and receptors, aiming to identify the active pockets. Finally, molecular docking simulations were conducted using Autodock Vina software, and the results were visualized with PyMol 2.4.0.

### 2.8. The mRNA Expression Levels of lptD and msbA in MDR AB 55282 Under the Action of Bioactive Components

To verify the above inferences, lptD and msbA were validated at the mRNA level through quantitative polymerase chain reaction (qPCR) analysis. Meanwhile, 16sRNA was used as an internal control, and the primers used for the target genes are listed in Table 2. Total RNA was extracted using Total RNA extraction (TIANGEN, Beijing, China), and cDNA synthesis (Sparkjade, Qingdao, China) of MDR AB 55282 with and without linarin/hyperoside was performed according to the manufacturer’s instructions. qPCR was carried out using SYBR Green Master Mix on a Real-Time PCR System. The reaction conditions were 94 °C for 3 min, followed by 40 cycles of amplification at 94 °C for 10 s and 60 °C for 60 s. The assays were repeated three times.

Each assay contained three replicates for all genes, and the relative fold changes were calculated using the 2^−ΔΔCt^ method, as described. The significance of the differences between mean values was determined with one-way ANOVA.

### 2.9. Statistical Analysis

Data were analyzed by GraphPad Prism 8.0 (GraphPad Software, San Diego, CA, USA). The statistical differences among the different groups were compared by one-way ANOVA, and Dunnett’s multiple comparisons were used to calculate *p* values. All values of *p* < 0.05 were considered statistically significant and are indicated in the table and figure legends: *: *p* < 0.05; **: *p* < 0.01; ***: *p* < 0.001.

## 3. Results

### 3.1. Results of the Sensitivity Assay of Clinically Isolated MDR AB in Commonly Used Clinical Antibacterial Agents

This research adhered to the standardized protocols promulgated by the Clinical and Laboratory Standards Institute (CLSI, 2024). Employing the microbroth dilution assay and the Kirby–Bauer (K-B) disk diffusion technique, we evaluated the in vitro activities of a panel of first-line clinical antimicrobials against clinically derived MDR AB isolates. The resistance frequencies of ten MDR AB strains to these antimicrobials are detailed in Table 3. The findings revealed that all ten MDR AB strains exhibited elevated resistance levels towards β-lactam, quinolone, and aminoglycoside classes. Notably, profound resistance was detected against β-lactams, the crucial classes of antibiotics in the clinical management of Gram-negative bacterial infections. This resistance pattern highlights the urgent need for alternative therapeutic strategies to combat MDR AB infections.

As delineated in Table 4, tigecycline, colistin, and minocycline exhibited susceptibility against MDR AB 55282, indicating their continued efficacy in antimicrobial activity. Conversely, the meropenem/vaborbactam, imipenem/relebactam, and ampicillin/sulbactam combinations demonstrated pronounced resistance profiles against MDR AB 55282, rendering them largely ineffective in inhibiting the growth of this strain. Additionally, agents including imipenem, meropenem, cefoxitin, compound trimethoprim, gentamicin, tobramycin, and amikacin manifested resistance, thereby limiting their therapeutic utility in the treatment of infections caused by MDR AB 55282.

### 3.2. Antimicrobial Activity of Colistin and Senecio scandens Against MDR AB

The MIC and MBC of colistin and *Senecio scandens* against MDR AB were determined using the microbroth dilution method. As depicted in Table 5, the MIC values of colistin for ten MDR AB strains ranged from 0.25 to 2 μg/mL, with corresponding MBC values spanning 0.5 to 4 μg/mL. Notably, six of these strains exhibited non-susceptibility to colistin. In contrast, senecio scandens demonstrated consistent MIC values of 640 μg/mL across all ten MDR AB isolates, while MBC values fluctuated within the 640–1280 μg/mL range. When juxtaposed with the antibacterial dosage of *Senecio scandens* (1950–7810 μg/mL) reported by Ao et al. [24], the concentrations employed in this investigation were substantially lower. This discrepancy strongly suggests that *Senecio scandens* possesses potent antibacterial activity against *A. baumannii*.

### 3.3. Time-Kill Assays

**Figure 2:**
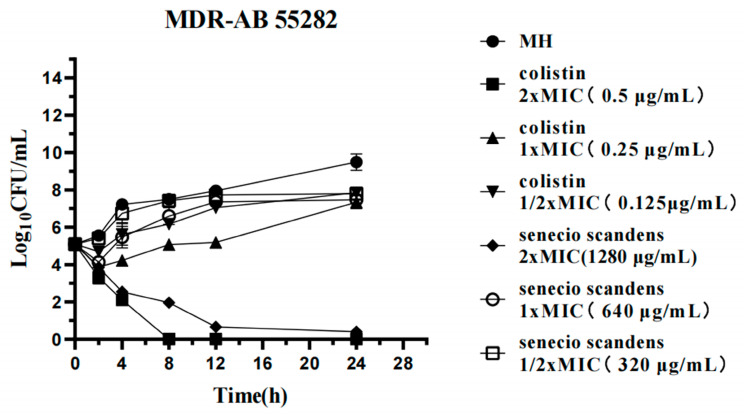
The time-kill assays of colistin or *Senecio scandens* at an initial concentration of 1.5 × 10^5^ CFU/mL was subsequently examined.

This investigation systematically evaluated the time-kill assays of MDR AB 55282. In the blank control group, the bacteria exhibited exponential growth, which strongly evidenced the strain’s rapid proliferation capacity under optimal environmental conditions. Upon exposure to 0.25 μg/mL of colistin, a significant reduction in bacterial load was observed during the initial 2 h interval. Although bacterial resurgence occurred after 4 h, the growth rate of the treated culture remained approximately 2 log_10_ CFU/mL lower than that of the control group over a 24 h period. In contrast, at a concentration of 0.5 μg/mL, colistin demonstrated continuous bactericidal activity against MDR AB, resulting in almost complete inhibition of bacterial growth by the eighth hour. These findings indicate that colistin exerts a potent bactericidal effect on MDR AB, with its efficacy exhibiting a clear concentration-dependent characteristic (Figure 2).

At a concentration of 320 μg/mL, senecio scandens did not exhibit any bactericidal effect. When the concentration was 640 μg/mL, it significantly reduced the initial bacterial quantity within the first 2 h. Subsequently, the bacteria grew slowly after 4 h, and its growth rate within 24 h was lower than that of the blank control group. This indicates that at this dosage, senecio scandens possessed mild bacteriostatic activity. Under the action of 1280 μg/mL of senecio scandens, the bacterial quantity was approximately 10^2^ at 8 h, which represented a decrease of 3 log_10_ CFU/mL compared to the situation with 640 μg/mL of senecio scandens at 8 h. The bacterial quantity approached zero after 12 h and remained at this low level until 24 h, demonstrating a strong bacteriostatic effect (Figure 2).

### 3.4. Ultrastructural Changes of MDR AB 55282 Induced by Senecio scandens

**Figure 3:**
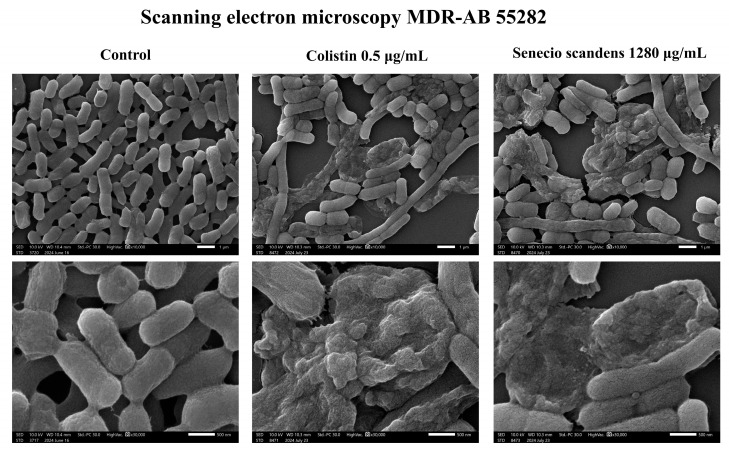
SEM results of MDR AB 55282 under the intervention of colistin or senecio scandens. The setup parameters for all SEM are as follows: (1) unified magnification (10,000×; 30,000×); (2) consistent scale bars (1 µm; 500 nm).

**Figure 4:**
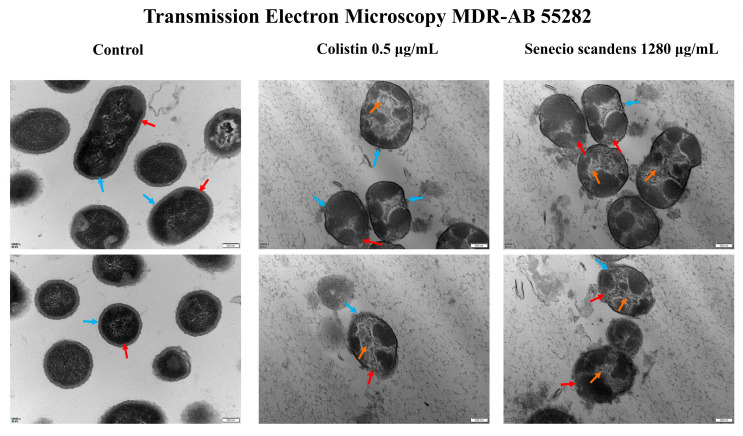
TEM results of MDR AB 55282 under the intervention of colistin or senecio scandens. The blue arrow represents the cell wall, the red arrow represents the cell membrane, and the orange represents cytoplasmic lysis. The setup parameters for all TEM are as follows: (1) unified magnification (60,000×); (2) consistent scale bars (200 nm).

The effects of colistin and senecio scandens on the morphology of MDR AB 55282 are illustrated in Figure 3 and Figure 4. As observed by scanning electron microscopy, the bacteria in the blank control group exhibited a plump morphology with regular shapes and uniform sizes. In contrast, after treatment with colistin and *Senecio scandens*, the bacterial count significantly decreased and the morphology shrank and became irregular. Under transmission electron microscopy, the bacteria in the blank control group displayed normal cellular morphological structures, presenting as rod-shaped with blunt ends. The cell walls had a uniform thickness, and the surface was relatively smooth, with a continuous and clear cell membrane. Both the colistin group and the senecio scandens group showed obvious abnormalities. The bacteria shrank and took on a round or oval shape. The cell walls dissolved and became thinner, and there were gaps in the cell walls and cell membranes in some areas, with the intracellular contents dissolving. The antibacterial mechanism of colistin has been demonstrated to primarily cause bacterial lysis and death by disrupting the integrity of the outer and inner membranes of bacteria [25]. Under the field of transmission electron microscopy, senecio scandens exhibited a destructive effect similar to that of colistin. Therefore, it is reasonable to consider whether senecio scandens exerts its corresponding bacteriostatic activity by disrupting the integrity of the cell membrane.

### 3.5. Effects of Colistin and Senecio scandens on Cell Membrane Integrity

**Figure 5:**
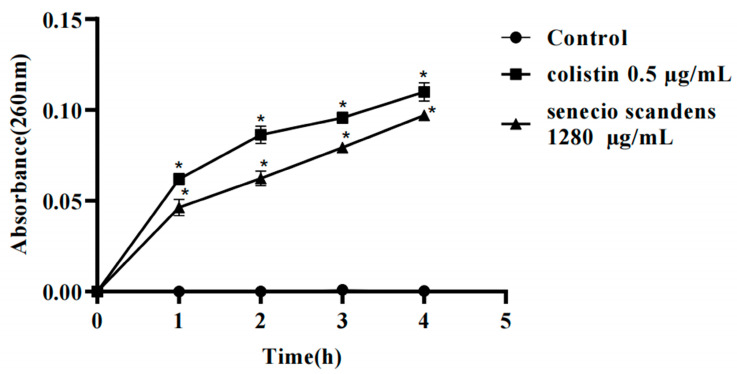
The release of intracellular components was examined in suspensions of MDR AB 55282 treated with COL (0.5 μg/mL) or *Senecio scandens* (1280 μg/mL). Denotes a significant difference compared to the control group (* *p* < 0.05). The data are presented as the mean ± standard deviation (*n* = 6).

The structural integrity of the bacterial cell membrane is fundamental for maintaining the homeostasis of intracellular components. According to previous studies, the presence of nucleic acid substances, including deoxyribonucleic acid (DNA) and ribonucleic acid (RNA), in the extracellular environment of bacteria typically indicates membrane disruption [26,27]. Additionally, extracellular DNA has been shown to be intricately involved in cell membrane biogenesis and contributes significantly to membrane stability [28]. This interplay underscores the pivotal role of nucleic acids in both assessing membrane integrity and supporting membrane functionality. Based on the results of TEM, the dissolution and leakage of intracellular substances in *A. baumannii* were observed. Hence, quantifying the release of intracellular nucleic acids can be regarded as an effective approach for evaluating the degree of cell membrane damage. The amounts of DNA and RNA released from *A. baumannii* cells in the suspensions treated with colistin and senecio scandens are depicted in Figure 5. After treatment with colistin and senecio scandens, nucleic acids within the bacteria leaked to different extents. Additionally, with the prolongation of the treatment time, the amount of leakage increased, and the most remarkable increase was observed at the time point of 4 h. In contrast, the OD values (at 260 nm) of the blank group showed almost no change. The results suggest that colistin kills *A. baumannii* by disrupting the cell membrane, subsequently leading to the leakage of intracellular DNA and RNA. This finding is consistent with previous reports stating that colistin exerts its antibacterial efficacy by compromising the integrity of the cell membrane [25]. It is worth noting that the group treated with senecio scandens exhibited a destructive effect highly similar to that of colistin. In previous studies, when CD-g-CS was applied to Staphylococcus xylosus and E. coli, comparable results were obtained [29]. Exposure to Pulsatilla scabiosaefolia induced rapid rupture of the cell membranes in methicillin-resistant *Staphylococcus epidermidis*, triggering a substantial and immediate release of intracellular DNA and RNA [23].

### 3.6. Collection of the Active Compounds in Senecio scandens

By consulting domestic and international literature databases, 58 active components of *Senecio scandens* were obtained. Among them, there were 7 flavonoids, 15 organic acids, 12 alkaloids, 18 volatile oils, 5 carotenoids, and 1 terpenoid compound (Supplementary Data S1).

### 3.7. Obtaining the Target Protein

Based on 4d label-free quantitative proteomics, a total of 2740 proteins and 17,743 unique peptides were identified. Based on a fold change of >2 or <1/2 and *p* < 0.05, 511 proteins were considered as differentially expressed proteins (DEPs). Among them, 35 DEPs were upregulated and 476 DEPs were downregulated (Supplementary File S1). These data suggest that proteomics can provide us with sufficient information on DEPs, enabling us to explore the potential antibacterial mechanisms or pathways of MDR AB 55282 under the stress of *Senecio scandens*.

**Figure 6:**
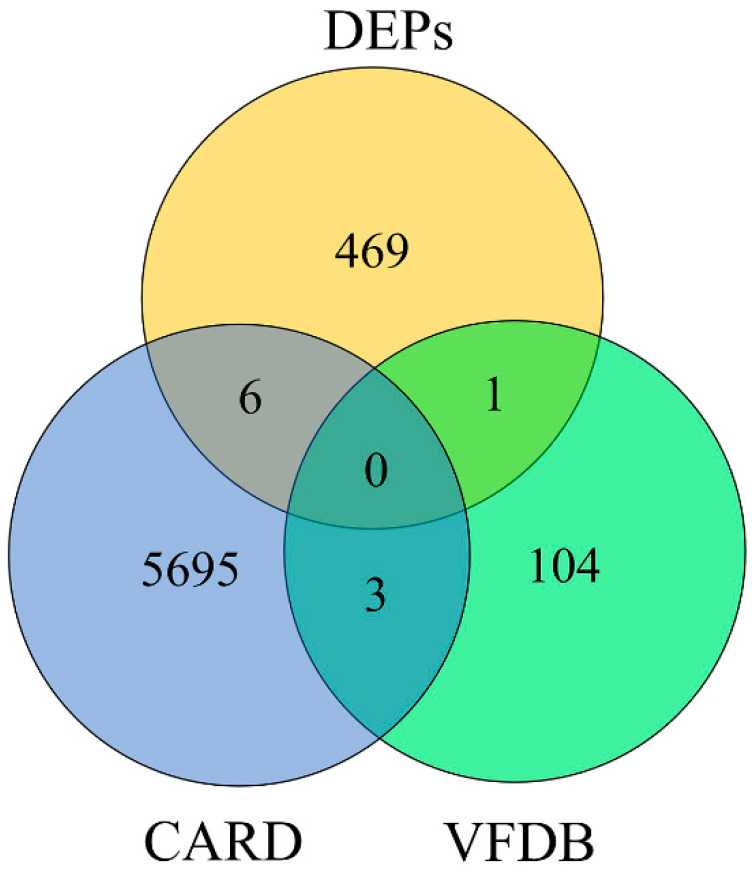
Venn diagram of downregulated DEPs, drug resistance proteins, and virulence proteins.

Through comparative analysis of the downregulated DEPs identified by proteomics with the VFDB and the CARD protein databases, an intersection dataset was generated to determine which of the downregulated DEPs were annotated in the databases, ensuring their relevance to virulence or antibiotic resistance mechanisms. One virulence protein (tssK) and six resistance proteins (catB8, carA, msbA, lptD, armA, and carO) were identified (Table 6; Figure 6). Among them, catB8 is a key gene for the resistance of *A. baumannii* to chloramphenicol [30], arma is a resistance gene for aminoglycoside antibiotics [31,32], carO is an outer membrane protein related to resistance to carbapenem antibiotics [33,34,35], lptD is an essential outer membrane protein in bacteria [36,37,38], and msbA plays a crucial role in the first important step of transporting lipopolysaccharide (LPS) from the inner membrane to the outer membrane [39,40].

### 3.8. Molecular Docking Between Active Components and Key Target Points

**Figure 7:**
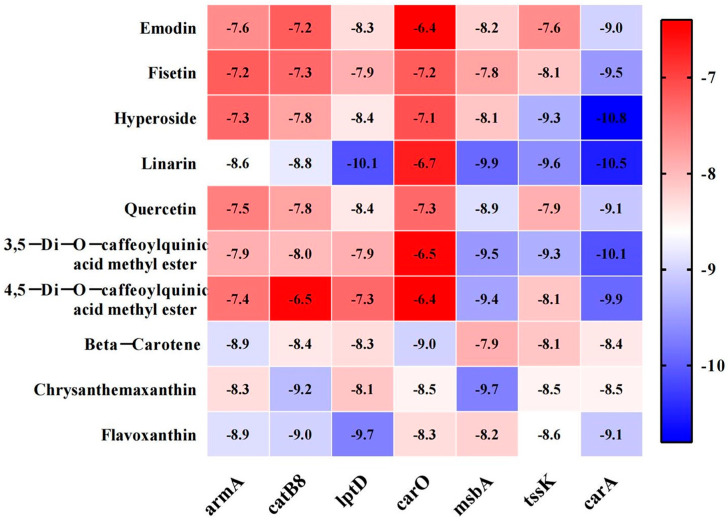
Heat map of molecular docking binding energy.

**Figure 8:**
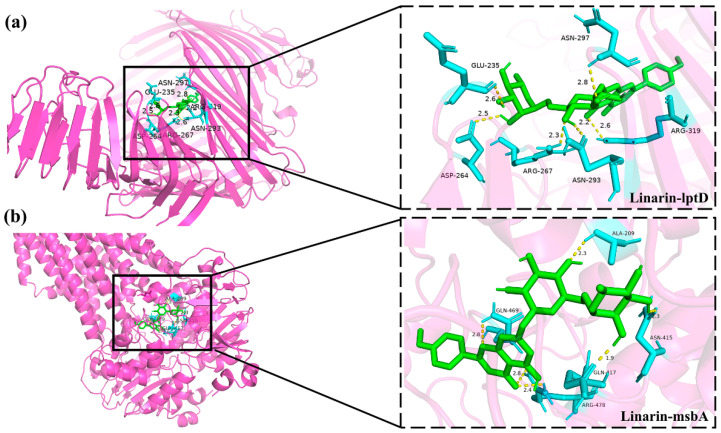
Molecular docking of bioactive components with key target sites: (**a**) linarin with lptD; (**b**) linarin with msbA. The molecule is represented by a green ball-and-stick model, while the amino acids are depicted by a blue ball-and-stick model.

**Figure 9:**
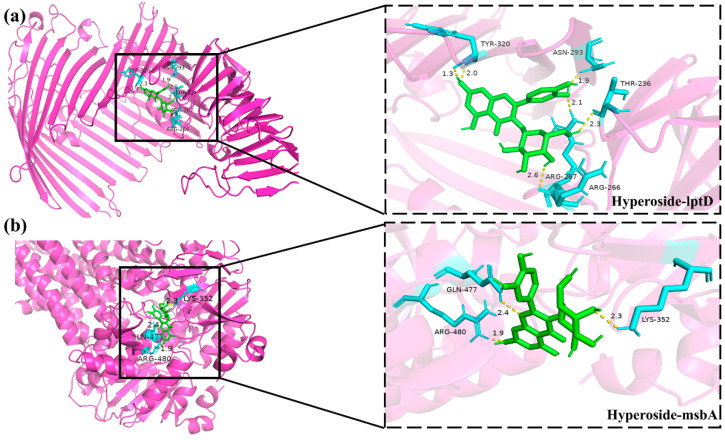
Molecular docking of bioactive components with key target sites: (**a**) hyperoside with lptD; (**b**) hyperoside with msbA. The molecule is represented by a green ball-and-stick model, while the amino acids are depicted by a blue ball-and-stick model.

Molecular docking experiments were performed to evaluate the binding potential between the active compounds derived from senecio scandens and seven hub proteins (tssK, catB8, carA, msbA, lptD, armA, and carO). Binding affinity, defined as the strength of interaction between a ligand and its receptor, was quantified using docking scores, where more negative values indicate stronger binding capabilities. Integrative analysis of electron microscopy findings and cell membrane integrity data strongly suggests that senecio scandens exerts its bactericidal activity by compromising cell membrane integrity. The msbA protein, a pivotal factor in the translocation of lipopolysaccharide (LPS) to the outer membrane [39], plays an indispensable role in maintaining membrane homeostasis; its deficiency has been shown to induce membrane perturbation and subsequent cell death [40]. Similarly, lptD, which encodes an essential outer membrane protein and mediates the transport of synthesized LPS to the outer membrane, is critical for preserving membrane integrity. Disruption of lptD has been demonstrated to lead to significant impairment of the cell membrane structure [36]. LPS exerts a pivotal influence on the development of biofilms in *A. baumannii* by modulating multiple key processes. It significantly contributes to the initial stage of microbial adhesion, which is a critical step for biofilm formation [41]. Consequently, the binding interactions between msbA, lptD, and the active constituents of senecio scandens emerge as key focal points for elucidating the underlying antibacterial mechanisms. Figure 7 illustrates the binding energies of ten active components that exhibit relatively strong binding capabilities with seven target proteins. The research findings indicate that linarin demonstrates favorable docking effects with lptD and msbA, with corresponding binding energies of −10.1 and −9.9 kcal/mol, respectively. Hyperoside also shows good performance, with binding energies of −8.4 and −8.1 kcal/mol for its interactions with lptD and msbA, respectively. However, the binding energies of colistin with LptD and MsbA are −6.1 kcal/mol and −5.9 kcal/mol, respectively. The molecular docking model is presented in the form of a 3D diagram using PyMol 2.4.0. Figure 8 shows the interactions between linarin and msbA as well as lptD, while Figure 9 demonstrates the interactions between hyperoside and msbA and lptD. Additionally, a binding pocket residue table has been added as Table 7.

### 3.9. Results of Antibacterial Activity Assays of Active Compounds Against MDR AB 55282

Since there were no matching compounds found for four bioactive components— namely, 3,5-Di-*O*-caffeoylquinic acid methyl ester, 4,5-Di-*O*-caffeoylquinic acid methyl ester, chrysanthemaxanthin, and flavoxanthin—only the in vitro antibacterial activities of six active components against MDR AB 55282 were determined. The results are shown in Table 8. The MICs of emodin, fisetin, quercetin, and β-carotene against MDR AB 55282 were all 5000 μmol/L, and the MBCs were all greater than 5000 μmol/L, indicating that these four inhibitors had no inhibitory activity against MDR AB. The MICs of linarin and hyperoside against MDR AB 55282 were both 312.5 μmol/L, and the MBCs were both 625 μmol/L, suggesting that these two inhibitors exhibited significant inhibitory activity against MDR AB 55282. The results demonstrated that linarin and hyperoside were the major active components of senecio scandens against *A. baumannii*.

**Figure 10:**
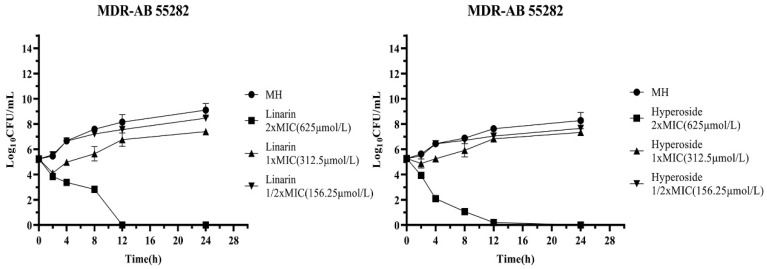
The time-kill assays of linarin and hyperoside at an initial concentration of 1.5 × 10^5^ CFU/mL were subsequently examined.

In this study, the time-kill curves under the action of linarin and hyperoside as individual inhibitors were determined. Linarin (CAS No. 480-36-4, and purity: ≥98.0%) and hyperoside (CAS No.: 482-36-0, and purity: 99.50%) were obtained from MedChemExpress, Shanghai, China. The bacterial quantity in the blank control group exhibited exponential growth in a suitable environment. When the linarin inhibitor was at a concentration of 312.5 μmol/L, the bacterial quantity was significantly reduced within the first 2 h. After 4 h, the bacterial quantity began to gradually increase, but the growth rate was distinctly lower than that of the blank control group. Under the action of 625 μmol/L linarin, the colony count of MDR AB 55282 significantly decreased. Almost no colonies of MDR AB 55282 were observed after 12 h, and there was no growth phenomenon until 24 h. When the hyperoside inhibitor was at a concentration of 312.5 μmol/L, the bacterial quantity was slightly reduced within the first 2 h. After 4 h, the bacterial quantity started to gradually increase, and after 8 h, its growth rate was on par with that of the blank control. Under the action of 625 μmol/L of hyperoside, the bacterial quantity was continuously and significantly reduced. The logarithm of the colony-forming unit value of MDR AB 55282 that could be detected after 12 h was close to 0, and there was no bacterial growth within 24 h (Figure 10).

### 3.10. Results of the mRNA Expression Levels of lptD and msbA in MDR AB 55282 Affected by Active Components

**Figure 11:**
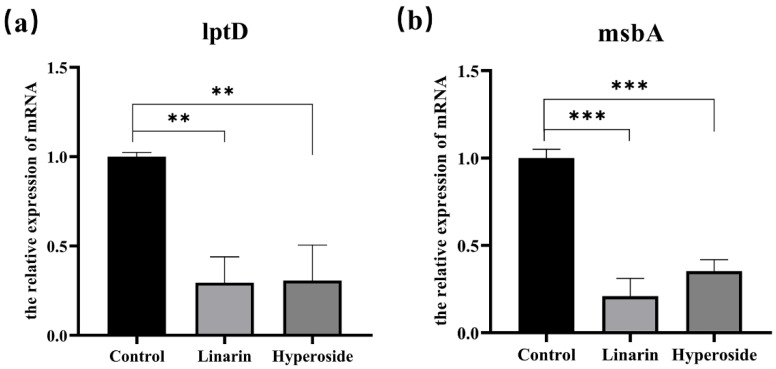
Effect of linarin and hyperoside on the transcription levels of MDR AB 55282 gene lptD and msbA. (**a**) Effect of linarin and hyperoside on the transcription levels of MDR AB 55282 gene lptD. (**b**) Effect of linarin and hyperoside on the transcription levels of MDR AB 55282 gene msbA. Significance levels compared to the blank control group: ** *p* < 0.01, *** *p* < 0.001.

The qPCR method was employed to examine the impacts of two bioactive components—namely, linarin and hyperoside—on the transcriptional levels of the lipopolysaccharide assembly protein lptD and the lipid transport ATPase msbA in MDR AB 55282. As presented in Figure 11, when compared with the blank control group, the expression levels of the gene lptD in both the linarin group and the hyperoside group were notably reduced (*p* < 0.01). Moreover, the expression level of the gene msbA significantly declined (*p* < 0.001), and these differences were statistically significant.

This research adhered to the standardized protocols promulgated by the Clinical and Laboratory Standards Institute (CLSI, 2024). Employing the microbroth dilution assay and the Kirby–Bauer (K-B) disk diffusion technique, we evaluated the in vitro activities of a panel of first-line clinical antimicrobials against clinically derived MDR AB isolates. The resistance frequencies of ten MDR AB strains to these antimicrobials are detailed in Table 3. The findings revealed that all ten MDR AB strains exhibited elevated resistance levels towards β-lactam, quinolone, and aminoglycoside classes. Notably, profound resistance was detected against β-lactams, the crucial classes of antibiotics in the clinical management of Gram-negative bacterial infections. This resistance pattern highlights the urgent need for alternative therapeutic strategies to combat MDR AB infections.

## 4. Discussion

The emergence of *A. baumannii* as a significant nosocomial pathogen has shocked the world and poses a major threat to the global healthcare system. Due to its capability to evade the effects of antibiotic drugs and exhibit a high level of antimicrobial resistance, it often leads to treatment failure [42,43]. In particular, its resistance to last-resort β-lactam antibiotics such as carbapenems (e.g., imipenem, meropenem) is especially concerning [44,45] and has become a significant challenge in the global healthcare field. As *A. baumannii* infections escalate as a worldwide health concern, intensifying clinical management demands, the imperative for innovative therapeutic approaches has never been greater.

This study systematically investigated the antibacterial activity of *Senecio scandens* against WHO-critical multidrug-resistant Acinetobacter baumannii (MDR AB), revealing key insights into its efficacy and mechanisms. MIC/MBC assays showed *S. scandens* exerted potent activity against all tested MDR AB strains (MIC = 640 μg/mL), a significantly lower and more efficient concentration than previously reported. In contrast, colistin—a last resort for MDR AB—exhibited variable susceptibility, with 6/10 strains non-susceptible. Time-kill curves confirmed concentration-dependent bactericidal effects: 1280 μg/mL nearly eliminated MDR AB within 12 h, while lower concentrations retained inhibition.

Ultrastructural (SEM/TEM) and cell membrane integrity analyses indicated both *S. scandens* and colistin induced morphological damage (shrinkage, irregularity, cell wall/membrane dissolution) [46] and disrupted membrane integrity—evidenced by intracellular nucleic acid release—consistent with a shared mechanistic step.

Proteomic analysis of *S. scandens*-treated MDR AB identified 511 differentially expressed proteins (476 downregulated), including one virulence- (tssK) and six resistance-associated proteins (e.g., msbA, lptD), with msbA and lptD critical for outer membrane integrity, aligning with observed membrane disruption [39,40,47,48]. Molecular docking showed that the *S. scandens* active components linarin and hyperoside strongly bound msbA and lptD, while their MIC/MBC against MDR AB 55282 and qPCR data (significant downregulation of lptD and msbA mRNA) further supported antibacterial potency via interference with membrane-associated gene expression.

Linarin and hyperoside are both flavonoids with extensive biological activities, particularly prominent in liver protection, anti-inflammation, and antioxidant effects. In this study, both linarin and hyperoside exhibited significant antibacterial activity. Due to poor water solubility and lipid solubility, linarin has low oral bioavailability (0.47%), but its pharmacokinetic properties can be improved through dosage form optimization, with favorable safety profiles [49,50,51]. Hyperoside also has low oral bioavailability, while absorption via intraperitoneal or intravenous injection is more efficient; attention should be paid to nephrotoxicity associated with long-term high-dose administration [52,53]. The clinical application potential of both compounds requires further verification through clinical trials.

In conclusion, this study provides comprehensive evidence that senecio scandens exerts antibacterial activity against MDR AB primarily by disrupting the cell membrane. Linarin and hyperoside are identified as key active components, and their interactions with msbA and lptD may play a central role in this process. These findings not only enhance our understanding of the antibacterial mechanisms of senecio scandens but also offer potential leads for the development of novel antibacterial agents against MDR AB. However, further in vivo studies and investigations into the safety profiles of senecio scandens and its active components are warranted to translate these findings into clinical applications.

## 5. Conclusions

This study reveals for the first time the molecular mechanism by which senecio scandens, a medicinal herb in Miao ethnic medicine, exerts its antibacterial effect. Specifically, its active components, linarin and hyperoside, target and inhibit the expression of lptD and msbA genes in MDR AB, disrupting the structural integrity of the bacterial outer membrane. Since the bacterial outer membrane is a crucial barrier for *A. baumannii* against external threats, its structural disruption enables other antibacterial substances to penetrate more effectively, thus enhancing the antibacterial activity. These findings not only deepen our understanding of the antibacterial mechanism of senecio scandens but also provide a theoretical basis for the development of novel drugs against MDR AB. Linarin and hyperoside, as the key active components, are promising candidate compounds for the development of new drugs, offering potential strategies to combat the challenges posed by MDR AB in clinical settings.

## Figures and Tables

**Table 1:** Sources and basic information of *Acinetobacter baumannii*.

Identification	No.	Gender	Department	Infection Source	Clinical Diagnosis
*Acinetobacter baumannii*	55282	Male	ICU	Sputum	Severe Pneumonia
*Acinetobacter baumannii*	61759	Female	ICU	Blood	Diabetic foot
*Acinetobacter baumannii*	64295	Male	Geriatrics	Secretion	Pressure ulcer
*Acinetobacter baumannii*	63440	Male	Respiratory medicine	Sputum	Severe Pneumonia
*Acinetobacter baumannii*	65912	Male	Hematology	Sputum	Lymphadenectasis
*Acinetobacter baumannii*	58195	Female	Orthopedics department	Drainage fluid	Multiple myeloma
*Acinetobacter baumannii*	55149	Female	ICU	Sputum	Renal insufficiency
*Acinetobacter baumannii*	55547	Male	ICU	Sputum	Severe Pneumonia
*Acinetobacter baumannii*	61412	Female	Geriatrics	Blood	Gastrointestinal hemorrhage
*Acinetobacter baumannii*	55731	Male	ICU	Sputum	Severe Pneumonia

**Table 2:** Primer sequences.

Gene	Primer 5′-3′
lptD	Forward: ACACCAGCAGCCTTTGTAATTCC
	Reverse: GAACCGCCGTCTAAACCTTGAG
msbA	Forward: TTTATCGGGTGGTCAACGTCAAC
	Reverse: CGCACTTGTCGCCTCATCC
16S rDNA	Forward: ACGACTTCACCCCAGTCATC
	Reverse: CACACCATGGGAGTTTGTTG

**Table 3:** Analysis of drug resistance of 10 MDR AB strains to 13 clinical first-line antimicrobials.

Type	Antibacterial Drug	Number of Cases (n)	Resistance Rate (%)
β-lactamase	Ampicillin/Sulbactam	6	60
	Ceftriaxone	8	80
	Piperacillin/tazobactam	2	20
	Ceftazidime	8	80
	Cefepime	7	70
	Cefazolin	5	50
	Imipenem	8	80
	Meropenem (K-B method)	5	50
Quinolones	Ciprofloxacin	6	60
	Levofloxacin	7	70
Aminoglycosides	Gentamicin	5	50
	Tobramycin	6	60
	Amikacin (K-B method)	8	80

**Table 4:** Susceptibility results of MDR AB 55282 to clinical first-line antibiotics.

Antibacterial Drug	MIC (μg/mL)	Determination Result	Antibacterial Drug (K-B Method)	Zone of Inhibition (mm)	Determination Result
Ceftazidime	>256	R	Amikacin	6	R
Meropenem/Vaborbactam	>256	R	Imipenem	9	R
Imipenem/Relebactam	>32	R	Ampicillin/Sulbactam	6	R
Meropenem	≥32	R	Cefoperazone/Sulbactam	18	I
Tigecycline	0.25	S	Minocycline	16	S
Colistin	0.25	S	Tigecycline	16	S
Cefoxitin	256	R			
Compound trimethoprim	≥320	R			
Gentamicin	≥16	R			
Tobramycin	≥16	R			

S: susceptive; I: intermediary; R: resistant.

**Table 5:** The MIC and MBC values of COL or Senecio scandens against MDR AB.

Strains	Colistin		*Senecio scandens*	
	MIC (μg/mL)	MBC (μg/mL)	MIC (μg/mL)	MBC (μg/mL)
55282	0.25	0.5	640	1280
61759	2	4	640	1280
64295	2	4	640	1280
63440	2	4	640	1280
65912	1	2	640	1280
58195	2	4	640	1280
55149	2	4	640	640
55547	0.5	1	640	640
61412	1	2	640	1280
55731	2	4	640	1280
ATCC 19606	0.25	0.25	640	1280

colistin > 2 μg/mL, determined to be resistant.

**Table 6:** Overlapping DEPs in proteomics, drug resistance gene databases, and virulence factor databases.

PDB ID	Protein ID	Gene Name	Fold Change of *Senecio scandens*/Control	*t* Test *p* Value
8J40	Q5D169	catB8	0.1761	0.00008
1T36	A0A009HU80	carA	0.1056	0.00005
9CSI	A0A009IKL5	msbA	0.0863	0.0003
4Q35	A0A219CCC6	lptD	0.1592	0.0075
3FZG	A7U830	armA	0.3681	0.0298
4FUV	Q4A209	carO	0.1511	0.0269
5M2Y	A0A059ZI91	tssK	0.2426	0.007

**Table 7:** Molecular docking results of linarin/hyperoside with lptD/msbA.

Compound Name	Gene Name	Docking Score (kcal/mol)	Hydrogen Bond
linarin	lptD	−10.1	GLU-235
			ASN-297
			ARG-319
			ASN-293
			ARG-267
			ASP-264
	msbA	−9.9	ALA-209
			ASN-415
			GLN-417
			ARG-478
			GLN-469
hyperoside	lptD	−8.4	TYR-320
			ASN-293
			THR-236
			ARG-266
			ARG-267
	msbA	−8.1	GLN-477
			LYS-352
			ARG-480

**Table 8:** Determination of MIC and MBC concentrations of six bioactive components against MDR AB.

Bioactive Components	Molecular Weight (g/mol)	Chemical Formula	MIC		MBC	
			μmol/L	g/L	μmol/L	g/L
Emodin	270.23	C_15_H_10_O_5_	5000	1.35	>5000	>1.35
Fisetin	286.23	C_15_H_10_O_6_	5000	1.43	>5000	>1.43
Hyperoside	464.38	C_21_H_20_O_12_	312.5	0.15	625	0.29
Linarin	592.55	C_25_H_32_O_14_	312.5	0.19	625	0.37
Quercetin	302.23	C_15_H_10_O_7_	5000	1.51	>5000	>1.51
β-carotene	536.88	C_40_H_56_	5000	2.68	>5000	>2.68

## Data Availability

The raw data supporting the conclusions of this article will be made available by the authors on request.

## Supplementary Materials

The following supporting information can be downloaded at: https://www.mdpi.com/article/10.3390/biology14081087/s1, Supplementary Data S1: Supporting Information; Supplementary File S1: Differentially Expressed Proteins.

## Abbreviations

The following abbreviations are used in this manuscript:

MDR AB	multidrug-resistant *Acinetobacter baumannii*
MIC	minimum inhibitory concentration
MBC	minimum bactericidal concentration
SEM	scanning electron microscopy
TEM	transmission electron microscopy
