# Supplementary material for: Linarin and Hyperoside Inhibit lptD/msbA to Disrupt Membranes of Multidrug-Resistant Acinetobacter baumannii
Source: Biology (Basel). 2025 Aug 20;14(8):1087. doi: 10.3390/biology14081087 (PMC12383518; doi:10.3390/biology14081087)
Supplement: Supplementary file 1 [file biology-14-01087-s001.zip › Supplementary Data S1.pdf]

SUPPORTING INFORMATION

Data S1 The active components in sencio scandens.

Type	Active components	Chemical formula	CID number
Flavonoids	quercetin	$C_{15}H_{10}O_7$	5280343
	Hyperoside	$C_{21}H_{20}O_{12}$	5281643
	Kaempferol	$C_{15}H_{10}O_6$	5280863
	Linarin	$C_{28}H_{32}O_{14}$	5317025
	emodin	$C_{15}H_{10}O_5$	3220
	Isorhamnetin	$C_{16}H_{12}O_7$	5281654
	Fisetin	$C_{15}H_{10}O_6$	5281614
Organic acids	Caffeic Acid	$C_9H_8O_4$	689043
	Ethyl caffeate	$C_{11}H_{12}O_4$	5317238
	P-Coumaric acid	$C_9H_8O_3$	637542
	4,5-Di-O-caffeoylquinic acid methyl ester	$C_{26}H_{26}O_{12}$	10052718
	3,5-Di-O-caffeoylquinic acid methyl ester	$C_{26}H_{26}O_{12}$	10075681
	3,4-Di-O-caffeoylquinic acid methyl ester	$C_{26}H_{26}O_{12}$	10392218
	Chlorogenic Acid	$C_{16}H_{18}O_9$	1794427
	2,3-Dihydroxypropyl hexadecanoate	$C_{19}H_{38}O_4$	14900
	Hydroquinone	$C_6H_6O_2$	785
	(p-Hydroxyphenyl)acetic acid	$C_8H_8O_3$	127
	Vanillic Acid	$C_8H_8O_4$	8468
	Salicylic acid	$C_7H_6O_3$	338

Data S1 Continued.

	Pyromucic acid	$C_5H_4O_3$	6919
	2-(1,4-Dihydroxycyclohexyl)acetic acid	$C_8H_{14}O_4$	54033324
	dictamnoside A	$C_{21}H_{36}O_9$	44560015
alkaloids	Neoplatyphylline	$C_{18}H_{27}NO_5$	6912281
	Senecionine	$C_{18}H_{25}NO_5$	5280906
	Senecionine N-oxide	$C_{18}H_{25}NO_6$	5380876
	Seneciphylline	$C_{18}H_{23}NO_5$	5281750
	SeneciphyllineN-oxide	$C_{18}H_{23}NO_6$	13258912
	Senkirkine	$C_{19}H_{27}NO_6$	5281752
	Jacobine	$C_{18}H_{25}NO_6$	442741
	jacobine N-oxide	$C_{18}H_{25}NO_7$	132282048
	Monocrotaline	$C_{16}H_{23}NO_6$	9415
	Adonifoline	$C_{18}H_{23}NO_7$	15736564
	Hordenine	$C_{10}H_{15}NO$	68313
	Usaramine	$C_{18}H_{25}NO_6$	5281756
Volatile oils	1-Tetradecene	$C_{14}H_{28}$	14260
	4-Vinylphenol	C_8H_8O	62453
	2-Methoxy-4-vinylphenol	$C_9H_{10}O_2$	332
	Camphene	$C_{10}H_{16}$	6616
	(E,E)-alpha-farnesene	$C_{15}H_{24}$	5281516
	Tricyclene	$C_{10}H_{16}$	79035
	Alpha-caryophyllene	$C_{15}H_{24}$	5281520
	(-)-Caryophyllene oxide	$C_{15}H_{24}O$	1742210
	Palmitic acid	$C_{16}H_{32}O_2$	985
	Linoleic Acid	$C_{18}H_{32}O_2$	5280450

Data S1 Continued.

	Linalool	$C_{10}H_{18}O$	6549
	Alpha-Terpineol	$C_{10}H_{18}O$	17100
	Geraniol	$C_{10}H_{18}O$	637566
	Borneol	$C_{10}H_{18}O$	64685
	Eugenol	$C_{10}H_{12}O2$	3314
	p-cymene	$C_{10}H_{14}$	7463
	Phytantriol	$C_{20}H_{42}O_3$	3018525
	1-Methyl-2-Pentylcyclopropane	C_9H_{18}	524612
	alpha-Carotene	$C_{40}H_{56}$	6419725
	Beta-Carotene	$C_{40}H_{56}$	5280489
Carotenoids	beta-Zeacarotene	$C_{40}H_{58}$	5280790
	Chrysanthemaxanthin	$C_{40}H_{56}O_3$	21160900
	Flavoxanthin	$C_{40}H_{56}O_3$	5281238
Terpenoid	Jacaranone	$C_9H_{10}O_4$	73307
